# The influence of participation in target-shooting sport for children with inattentive, hyperactive and impulsive symptoms – A controlled study of best practice

**DOI:** 10.1186/s12888-017-1283-5

**Published:** 2017-03-28

**Authors:** Annegrete Gohr Månsson, Mette Elmose, Søren Dalsgaard, Kirsten K. Roessler

**Affiliations:** 1 Department of Psychology University of Southern Denmark, Campusvej 55, -5230 Odense, DK Denmark; 2 National Centre for Register-based Research, Aarhus University, Aarhus, Denmark; 3 The Lundbeck Foundation Initiative for Integrative Psychiatric Research, iPSYCH, Aarhus, Denmark; 4 Department for Child and Adolescent Psychiatry, Hospital of Telemark, Kragerø, Norway

**Keywords:** Children, ADHD, Hyperactivity, Inattentiveness, Impulsivity, Physical activity, Target-shooting sport, Mental training

## Abstract

**Background:**

Practising target-shooting sport requires focused attention and motoric steadiness. A previous non-controlled pilot study suggests that children with impairing symptoms of attention-deficit/hyperactivity disorder (ADHD) benefit from participating in target-shooting sport in local shooting associations, as rated by parents and teachers.

This study aims at examining if, and to which extent, target-shooting sport reduces parent- and teacher-reported severity of inattentiveness, hyperactivity, and impulsivity in children with attention difficulties, and if, and to which extend, target-shooting sport improves the children’s wellbeing and quality of life.

**Methods:**

A mixed method approach is applied. A non-blinded, waiting list controlled study is combined with a case study, consisting of interviews and observations. The intervention consists of children practising target-shooting sport, by attending a local shooting association, once a week for six months, during regular school hours. Data from questionnaires (ADHD-RS, SDQ, Kidscreen-27), as well as a computerized continued performance test (Qb test), measure the children’s activity and attention. The study includes 50 children in an intervention group and 50 children in a waiting list control group. The Qb test collects data from at least 20 children from the intervention group and at least 20 children from the waiting list control group. Data from the questionnaires and Qb-test is collected at baseline, and six months post intervention. In addition, a case study is carried out, consisting of interviews of at least five children from the intervention group, their parents, teachers and shooting instructors. Observations are carried out, when children are in school and while they are attending the local shooting association. The case study adds to an in-depth understanding of children’s participation in target-shooting sports.

**Discussion:**

At present, little is known about the effects and influence of practising target-shooting sport for children experiencing difficulties with inattentiveness, hyperactivity and impulsivity. This study is expected to contribute to an understanding of the influence of participating in target-shooting sports on inattentive, hyperactive and impulsive symptoms, and the effects on the children’s psychological wellbeing and quality of life.

**Trial registration:**

Current Controlled Trials NCT02898532. Retrospectively registered 14 September 2016.

## Background

Children diagnosed with attention-deficit/hyperactivity disorder (ADHD) (DSM-5) are characterized by three core symptoms: inattention, hyperactivity and impulsivity [1]. Approximately 2–3% of children attending Danish schools are diagnosed with ADHD [2, 3], and worldwide the population prevalence of ADHD is 5% [4]. Many children with ADHD experience difficulties in managing social relations, and are often excluded from participating in leisure activities with other children, e.g. team sports [5]. This may have negative impacts on their wellbeing and quality of life, which depend on having friends and relationships with peers [5]. Between 25 and 50% of those with childhood ADHD experience persisting symptoms and impairment in adulthood, and many develop psychological and social problems [6] and are at risk of premature death [7]. The NICE guideline [8] recommends a combination of pharmacological and psychosocial treatment (multimodal treatment approach) for children aged 6–18 years, to remedy symptoms of both ADHD and comorbid disorders. A growing focus on the benefits of sports activities, yoga and mindfulness suggests that physical activity may have a positive impact on children and young people, also those with ADHD [9–13].

Target-shooting sport may be regarded as mental training, where the participant uses breathing techniques to calm down and focus, and thereby improves attention. Mind and body must be in complete balance during practice. These techniques are somewhat similar to those used in meditation [14]. The Danish Shooting Association has nationwide standards and regulations, which must be adhered to by all participants practising and instructing target-shooting sports. These clear guidelines and boundaries have several characteristics, which may be beneficial for children, with ADHD. While at the shooting range, the children practice in separate, individual cubicles. They use earmuffs to protect their hearing. Furthermore, each child receives guidance and supervision from an individual adult instructor. The instructor will be standing next to the child at all times. It is custom for the instructor to give instructions in a calm, structured and concise manner. Finally, when practising target-shooting sport, the children receive immediate feedback on their performance, in terms of whether they hit the target or not. Thus, target-shooting sports has per se elements, similar to elements in behavioural interventions often directed at children with ADHD or oppositional defiant disorder/conduct disorder. In addition, the sport has elements of mental training, which may contribute to reducing ADHD-symptoms, and comorbid symptoms, and participating in a sport without requiring additional help or guidance, compared to children without ADHD, may strengthen the child’s wellbeing and self-perceived quality of life. In 2012, DGI Shooting (A large sports organization in Denmark) initiated a sport project, where children with ADHD or sub-threshold symptoms of inattention, hyperactivity and impulsivity practiced target-shooting sports in local shooting associations, during regular school hours. The sport project was initiated as a collaboration between DGI Shooting, local shooting associations and local schools. The evaluation showed, that participating children increased their concentration-span and focus during the training, and the children had valuable experiences of success, when practising the sport. Some of the children also returned to the local shooting associations in their spare time, and thereby participated in leisure activities with children outside the sport project [15]. This study has been based on these preliminary findings. To the best of our knowledge, no previous controlled trial has investigated the effects and influence of practising target-shooting sport, for children with difficulties with inattention, hyperactivity and impulsivity. The main aim of this study is therefore to investigate the influence of participating in target-shooting sports in Danish Shooting Associations, as an intervention for children exhibiting difficulties with inattention, hyperactivity and impulsivity, on symptoms of ADHD and comorbid symptoms and on quality of life.

## Methods and design

**Fig. 1:**
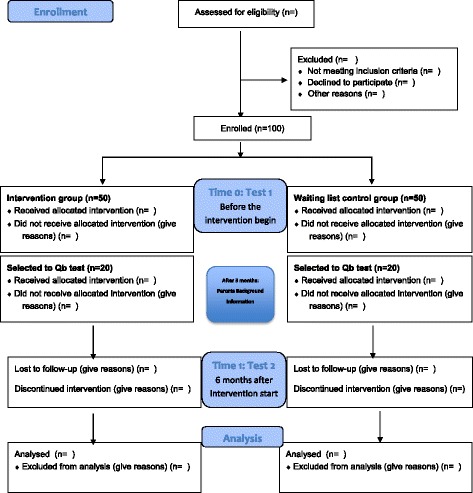
Flow Diagram (Inspired of CONSORT 2000)

A mixed method approach is used in a pre-post design [16] (Fig. 1) combining a case study [17] with a multiple informant survey and a computer test of the children’s performances.

### Objectives

The study will investigate beneficial and adverse effects of the intervention on children’s:

Inattention, hyperactivity, and impulsiveness.

Comorbid symptoms, such as emotional, social and behavioural difficulties.

Well-being and quality of life.

We hypothesise, that participation in the target-shooting sports in local Danish shooting associations, will reduce the children’s symptoms of inattention, hyperactivity and impulsivity, their comorbid symptoms, and enhance their wellbeing and self-reported quality of life.

### Participants

Inclusion: Children aged 10–14, meeting the following criteria: either a clinical diagnosis of ADHD or rated by school staff or school psychologist to have impairing difficulties with inattention, hyperactivity and impulsivity, affecting the child’s school attendance. Included schools are either, schools for children with special needs or municipal schools with special educational programmes for children diagnosed with either ADHD or severe and impairing difficulties of hyperactivity, inattention and impulsivity. If the child receives pharmacological treatment for ADHD, the treatment should not be changed (dosage, timing, class of drug), during the study period.

Exclusion: Children with visual impairment, current psychotic symptoms, suicidal ideations or behaviour are excluded from the study.

### Measures

*ADHD-RS* [18]: The questionnaire is parent- and teacher reported and is used to assess the severity of core symptoms of ADHD (inattention, hyperactivity, and impulsivity). The questionnaire is widely used to evaluate change before and after initiation of a treatment. The version translated into Danish is the ADHD-RS-IV, a modified version consisting of 26 items, reflecting three subscales: Inattention (9 items), Hyperactivity/impulsiveness (9 items) and behavioural difficulties (8 items) [19]. In this trial, the total score is based on the 18 items measuring inattention and hyperactivity/impulsiveness (total score of 0–54).

*SDQ - Strengths and Difficulties Questionnaire* [20]: The questionnaire consists of five subscales: 1) Hyperactivity/inattention, 2) Emotional issues, 3) Behavioural issues, 4) Issues in relation to peers 5) Social strengths. The version with 25 items for parents and teachers is applied [21].

*KIDSCREEN-27* [22]: The self-report questionnaire for children focuses on health-related quality of life in children and young people aged 8–18. The questionnaire consists of five dimensions: 1) Physical wellbeing (5 items), 2) Psychological well-being (7 items), 3) Autonomy and parental relations (7 items), 4) Friends and social support (4 items), and 5) School environment (4 items). The questionnaire consists of 27 statements. It is recommended that the children complete the questionnaire during regular school hours in the presence of a teacher, or other adult, with whom the child is familiar, so that he or she can assist the child, if necessary.

*Qb Test* [23]: The Qb Test is a computer-test combining infrared camera recordings of the child’s movements, with a concurrent computer test, a *Continued Performance Test,* which measures the child’s attention and impulse control. The Qb Test produces quantitative measurements of difficulties in the three areas, characterizing the core symptoms of ADHD; inattention, hyperactivity and impulsiveness. The test has previously been proven valid and sensitive, in the monitoring of the effect of pharmacological treatment of children and young people’s ADHD core symptoms [23, 24].

*Semi-structured interviews *[25, 26]: The interviews focus on the participants’ experiences, reflections and impressions, regarding the period of intervention, as well as everyday life, in order to assess the influence of target-shooting sport for children with inattentive, hyperactive and impulsive symptoms. The interviews follow manuals, developed by the research team for each target group (parents, children, school staff and target shooting sport instructors). All participants are encouraged to speak freely and raise additional issues of importance. All interviews are audiotaped and transcribed verbatim. The interviews are analysed, in accordance with the descriptive phenomenological psychological method [26].

*Observations* [25]: Observations of participating children will be carried out in schools and during practise at the local shooting association, The observations are videotaped, if possible. An observation sheet developed especially for this study is used to guide and structure the observations.

#### Outcomes

The primary outcome is:

- The total score on the 18 items on symptoms of inattention, hyperactivity and impulsivity on the teacher-rated ADHD-RS-IV.

Secondary outcomes are:

- Hyperactivity (distance and area) and inattention (reaction time variance and omission errors) as measured by the Qb Test.
- The total score on the relevant 18 items on symptoms of inattention, hyperactivity and impulsivity on the parent-rated ADHD-RS-IV.
- The total scores on the teacher- and parent-rated SDQ.
- Quality of life as measured by the total score on the child-rated Kidscreen-27.

Interviews and observations will add to a phenomenological understanding of children’s participation and engagement in their daily life and will qualify to an in-depth understanding of the mechanisms of change.

### The intervention

The intervention is organised in collaboration with DGI Shooting, local schools, local shooting associations and the University of Southern Denmark. The intervention is carried out nationwide in Denmark. The children attend the shooting range for about 1 h, practicing rifle shooting, once a week, for a period of six months. Teachers accompany the children to the local shooting associations, where their shooting instructors meet them. Before the shooting session there is formal introduction and preparation. Every child shoots approximately 20 to 30 min. As part of each training session, participating children meet up in the club’s common room after the shooting session, where the children receive feedback before going back to school, with their teachers.

### Safety procedures

Due to regulations in the Danish Shooting Associations, riffles are kept in a locked weapons storage cabinet, which requires two separate keys to open. Only members of the Shooting Association, who hold permission from the police, hold the keys for the weapons cabinet. The riffles are never allowed to leave the club area, and throughout the shooting practice an individual, responsible adult instructor will monitor and watch each child, at all times.

### Procedures

The participating schools identify, which children are eligible to enrol in the study. Participating children are not randomized, but included either to an intervention group or to a waiting list control group, as they are identified. Waiting list control children are recruited from schools that agree to participate in the study, but will not be part of the intervention until after study period. The children in the waiting list control group have the same characteristics as the children in the intervention group, but are not practicing target-shooting sport during the study period. Parents of all eligible children receive verbal and written information about the intervention, following the guidelines of the National Committee on Health Research Ethics. Upon parental consent, the teacher informs and provides written information to the child. The written information will be read out loud and explained to the child. Background information (including diagnosis, use of medication and participation in leisure time activities) are provided by parents one week prior to the intervention, three months into the intervention and 6 months post intervention. ADHD-RS-IV, SDQ, and KIDSCREEN-27 questionnaires are collected one week prior to the intervention. Data, from the waiting list control group, will be collected at the same time. Questionnaires will be distributed to parents and teachers. The children will fill out the questionnaire (Kidscreen-27) and complete the computer test (Qb Test) during regular school hours. A research assistant will carry out the Qb Test at the participating schools.

The multiple case study, combining interviews and observations, is nested in the quantitative survey adding substantial in-depth information. Children, parents, teachers and shooting instructors will be interviewed during the intervention. The interviews will mainly focus on experiences with the intervention and changes in quality of life and wellbeing for the children. Interviews with the children, teachers and parents will be carried out either at the school or at the child’s home address. Interviews with shooting instructors will take place in the local shooting associations. The first author will conduct and record all interviews.

### Sample size and statistical analyses

Sample size calculations are based on the following parameters: The total score of the first 18 items of ADHD-RS-IV is the primary outcome measurement. In a validation study of a Danish clinical sample of 10–17-year-old boys with ADHD, the mean total-score was 32.3 (standard deviation (SD) 10.2) [27]. With an effect of the intervention on this outcome with a 25% reduction in the total score (=24.2) and with an expected SD of 15.2), a power of 80%, 1:1 allocation in the two groups, two-sided testing and a significance level of 5%, a total sample of 78 is required. In an evaluation of the sport project preceding this study [15] the dropout rate from participation in target shooting sport was below 15%. Following these parameters and a more conservative expected dropout at 20% it is estimated that there should be 50 children enrolled in each group. The following online statistics programme has been used for the calculation: http://www.quantitativeskills.com/sisa/calculations/samsize.htm. The primary and secondary outcomes measured for both groups, pre- and post-intervention will be analysed using linear mixed-effects models (random coefficient models and multilevel models). With the mixed effects model approach, all available data will be used and intention-to-treat analyses applied. A thematic analysis [26] will be made separately for each case, followed by an analysis across all cases to investigate common themes and patterns [17]. The number of cases enrolled will depend on when data-saturation is reached.

## Discussion

Adapting the environment to provide a clear and concise structure, in regards to routines, time and space, is recognized as a helpful educational and method for children with ADHD symptoms [28, 29]. Developing a well-structured environment provides the child with an opportunity to improve self-control, regarding impulsivity and attention [29]. Children with ADHD tend to benefit from having their own protected physical space, when involved in focused activities. They also tend to benefit from having a designated adult close by, to provide support in retaining focus [29]. However, such beneficial effects are only possible, if the children find the activity interesting and not as a punishment and also, children with ADHD tend to be motivated by activities with an immediate feedback [24, 29, 30]. Earmuffs are often recommended for children with ADHD, to make a sound barrier, which eliminates auditory disturbances [28, 29].

All these above-mentioned elements are present, when practicing target-shooting sports in local shooting associations in Denmark. There are clearly defined, non-negotiable rules, a clear structure and a predictable setting. This makes target-shooting sport an activity, which is easy to understand and practice, also for children with ADHD. At the shooting range, participants are allocated an individual separate cubicle. Children are supervised at all times by a designated adult instructor, who stays in close proximity of the child. During practice the instructor intends to establish a good connection with the individual child, who benefits from support and recognition. When aiming and shooting, participants receive immediate feedback, as to whether they hit bulls-eye. Preliminary findings from the initial sport project indicate, that participating children enjoys target-shooting sport, show on-going motivation, while at the same time increasing their ability to stay focussed on hitting the target [15]. The unique characteristics of target-shooting sport, combined with theoretical knowledge on the subject of children with ADHD and preliminary project experience in the field, indicate beneficial effects of practising target-shooting sport in local Danish shooting associations in children with symptoms of inattentiveness, hyperactivity and impulsivity.

At present, no research has investigated the effects of target shooting sports in children with impairing symptoms of inattentiveness, hyperactivity and impulsivity. If found effective in this open trial, larger randomized controlled trials of this intervention are needed. If such trials provide evidence that practising target-shooting sports has beneficial effects, it may become a relevant supplement to the current interventions available for children with ADHD. This may aid the development and adaption of other leisure activities and physical activities and increase the possibilities of participation for children with ADHD. The results may further add to the continued search for effective and motivating non-pharmacological interventions that may help and support children with ADHD.
